# Human‐Specific Transcriptome of Ventral and Dorsal Midbrain Dopamine Neurons

**DOI:** 10.1002/ana.25719

**Published:** 2020-03-30

**Authors:** Jimena Monzón‐Sandoval, Ilaria Poggiolini, Tobias Ilmer, Richard Wade‐Martins, Caleb Webber, Laura Parkkinen

**Affiliations:** ^1^ Oxford Parkinson's Disease Centre University of Oxford Oxford; ^2^ UK Dementia Research Institute, Cardiff University Cardiff; ^3^ Nuffield Department of Clinical Neurosciences University of Oxford Oxford; ^4^ Department of Physiology Anatomy and Genetics University of Oxford Oxford United Kingdom

## Abstract

**Objective:**

Neuronal loss in the substantia nigra pars compacta (SNpc) in Parkinson disease (PD) is not uniform, as dopamine neurons from the ventral tier are lost more rapidly than those of the dorsal tier. Identifying the intrinsic differences that account for this differential vulnerability may provide a key for developing new treatments for PD.

**Methods:**

Here, we compared the RNA‐sequenced transcriptomes of ~100 laser captured microdissected SNpc neurons from each tier from 7 healthy controls.

**Results:**

Expression levels of dopaminergic markers were similar across the tiers, whereas markers specific to the neighboring ventral tegmental area were virtually undetected. After accounting for unwanted sources of variation, we identified 106 differentially expressed genes (DEGs) between the SNpc tiers. The genes higher in the dorsal/resistant SNpc tier neurons displayed coordinated patterns of expression across the human brain, their protein products had more interactions than expected by chance, and they demonstrated evidence of functional convergence. No significant shared functionality was found for genes higher in the ventral/vulnerable SNpc tier. Surprisingly but importantly, none of the identified DEGs was among the familial PD genes or genome‐wide associated loci. Finally, we found some DEGs in opposite tier orientation between human and analogous mouse populations.

**Interpretation:**

Our results highlight functional enrichments of vesicular trafficking, ion transport/homeostasis and oxidative stress genes showing higher expression in the resistant neurons of the SNpc dorsal tier. Furthermore, the comparison of gene expression variation in human and mouse SNpc populations strongly argues for the need of human‐focused omics studies. **ANN NEUROL 2020;87:853–868**

Parkinson disease (PD) results primarily from the loss of dopaminergic (DA) neurons from the substantia nigra pars compacta (SNpc), which is associated with both disease severity and duration.^1^ Currently, there are no therapeutic approaches that would slow down or stop these neurons from dying. The development of such disease‐modifying drugs will most likely only stem from understanding the molecular mechanisms underlying neurodegeneration in the human brain. Interestingly, not all DA neurons in SNpc are equally affected in PD, and several studies comparing neuronal densities between PD patients and healthy controls have shown that the ventral tier neurons are most susceptible to cell loss, whereas the dorsal tier neurons remain rather resistant in the early stages of PD.^2^, ^3^, ^4^, ^5^ The cell loss has been described to show a selective, temporospatial progression spreading from caudal to rostral, lateral to medial, and ventral to dorsal pattern.^2^ Thus, understanding the distinct molecular profiles of DA neurons from these different anatomic regions could help us to understand why certain neurons are more vulnerable than others. Moreover, therapeutic strategies aimed at modulating specific molecules in the vulnerable/resistant subtypes could protect against the progressive motor symptoms associated with the loss of DA neurons.

DNA microarrays have been the most frequently used technique by which numerous gene expression studies have compared the postmortem brain tissue of PD patients to healthy controls, with varying results (see Zheng et al^6^ for meta‐analysis). The majority of these studies have, however, used SNpc bulk tissue, where the subpopulations are not identified and the cellular composition is affected by the neurodegenerative process itself, including reactive astrogliosis and microglial activation. Their weakness is that they ultimately focus on downstream consequences of the disease process, rather than the underlying causes. Analysis of cell‐type–specific gene expression is possible with laser capture microscopy that allows the isolation of precise neuronal populations from the tissue.^7^ However, dissecting the rare surviving DA SNpc neurons remaining in end‐stage PD^8^, ^9^, ^10^ may only select cells more resistant to the disease process rather than revealing clues to the initial neurodegenerative mechanisms. Several studies have also compared transcriptomic profiles of SNpc and ventral tegmental area (VTA) in rodents,^11^, ^12^, ^13^ but how they relate to human gene expression is unknown. Up to 6 molecularly distinct subpopulations of SNpc and VTA were identified in mice by single‐cell gene‐expression profiling using microfluidic dynamic array on fluorescence‐activated cell sorting (FACS)‐sorted midbrain DA cells.^14^ Recently, these mouse subpopulations were also confirmed by RNA‐sequencing (RNA‐seq),^15^ which is superior to microarrays in both sensitivity and coverage.^16^

In the present study, for the first time in humans, we compared the RNA‐seq–derived transcriptomes of laser capture microdissected (LCM) ventral (vulnerable) and dorsal (resistant) tier neurons of the SNpc from healthy individuals, to gain insight into the intrinsic differences between these neuronal populations that could explain their differential vulnerability in disease. Understanding the molecular complexity of healthy midbrain DA neurons will help uncover novel candidates for a new generation of targeted therapeutic approaches to PD.

## Subjects and Methods

### Sample Collection and Preparation

**Figure 1:**
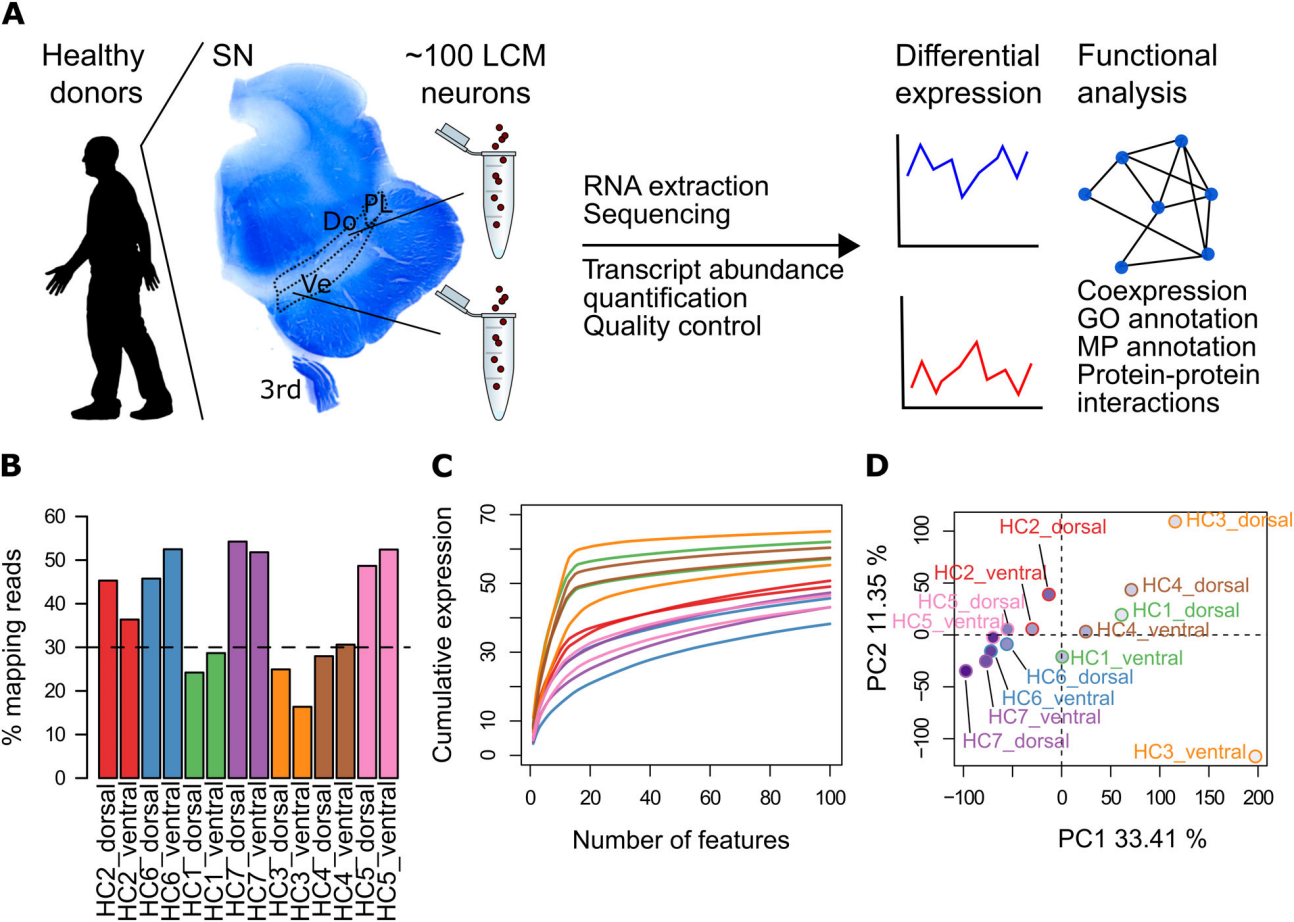
Protocol of workflow and quality control. (A) Protocol of workflow: from each healthy control donor, approximately ~100 neurons from the ventral (Ve) and dorsal (Do) tier of the substantia nigra (SN) were laser capture microdissected (LCM). Cells from 1 tier in each donor were pooled, RNA was extracted, and cDNA was obtained and prepared for sequencing, allowing us to compare the gene expression in vulnerable versus resistant neuronal populations and finally to perform functional analysis. 3rd = oculomotor nerve; GO = Gene Ontology; MP = Mammalian Phenotype; PL = pars lateralis. (B) Mapping rates as the percentage of reads mapping protein‐coding genes in each of the 14 available samples. The dashed line at 30% has been drawn to highlight samples with particularly lower mapping rates (healthy control [HC]1, 3, and 4 were excluded from further analysis). (C) Library complexity shown as the cumulative proportion of the library for the top 100 most expressed features/genes in each sample. (D) First 2 principal components (PC), which together explain ~45% of the variance. Label colors reflect the origin of the samples, whereas the intensity of the dots denotes the total number of counts in each sample (darker colors represent higher number of counts).

We extracted RNA from the frozen midbrain of ~30 healthy controls obtained from the Oxford MRC Control Brain Bank. South Central–Oxford C Research Ethics Committee approved the study (ethical license REC15/SC/0639). The RNA integrity number (RIN) was analyzed in these samples using Bioanalyzer (Agilent Technologies, Santa Clara, CA) and ranged between 6.6 and 9.2. Only 7 healthy controls with the highest RIN scores (≥8) were selected for the further analysis. The mean age at death in these 7 healthy controls (2 female, 5 male) was 70.7 ± 12.3 years (range = 56–93 years). Briefly, 10μm‐thick sections of the frozen midbrain were cut at the level of the 3rd nerve, dehydrated in ethanol series, and stained with cresyl violet in strictly RNA‐free conditions. Single neuromelanized neurons were isolated from separate, nonoverlapping ventral and dorsal tiers of SNpc using anatomic criteria and harvested from the stained cryosections by using a PALM Robot–Microbeam system (Carl Zeiss, Oberkochen, Germany; Fig 1).

### RNA Extraction and Sequencing Processing

In 7 healthy controls, the total RNA was extracted from ~100 pooled LCM neurons from each tier using PicoPure RNA isolation Kit (Arcturus, Mountain View, CA) with DNAse digestion step (RNAse‐Free DNAse Set; QIAGEN, Hilden, Germany). The cDNA was prepared for the sequencing using the SMARTer Kit (Clontech Laboratories, Mountain View, CA). A total of 14 samples, 2 from each tier of 7 healthy controls, were sequenced (HiSeq4000 75bp paired‐end) across 2 lanes. The resulting reads were mapped to cDNA sequences from *Homo sapiens* GRCh38 release 85 available through the Ensembl FTP site (ftp://ftp.ensembl.org/pub/). Kallisto version 0.42.4 was used to create an index and estimate counts and transcript abundances; default parameters were used except for the number of bootstraps, which was set to 100.^17^ Finally, the *tximport* function in R was used to summarize transcript abundances for all protein‐coding genes.

### Differential Expression

We used removal of unwanted variance (RUV) analysis to identify unwanted sources of variation.^18^ The first factor identified by RUV was found across a set of 1,000 empirical control genes and was found to be strongly correlated with the RIN score (*r* = 0.956). We incorporated the first factor identified into the model to test for differential expression between ventral and dorsal neurons using DESeq2.^19^ The model also considered the origin of the samples (ie, brain donor). Default filtering option was set up, and those genes with either a lower number of counts or outliers were filtered out. After filtering, 14,033 remained, and those were used as our background gene population for further analysis. Genes with a false discovery rate (FDR) < 0.05 were considered differentially expressed.

### TaqMan Reverse Transcribed Polymerase Chain Reaction Assay Validation

About 100 SNpc neurons were LCM in triplicates from each tier from the same healthy controls used for the RNA‐seq analysis. The total RNA was extracted using PicoPureTM RNA Isolation Kit (Applied Biosystems, Foster City, CA) from 3 LCM preparations for each tier per case and pooled during elution phase. This step was carried out to gain at least ~50ng of RNA, which was then reverse transcribed using SuperScript ViLO cDNA synthesis kit (Invitrogen, Carlsbad, CA). The obtained cDNA was assayed on a Rotor‐Gene Q Real‐Time PCR System (QIAGEN) using TaqMan Gene Expression Assays (Life Technologies, Carlsbad, CA) by following standard protocols (Table1). Reactions were performed in duplicates using the FAM dye‐labeled assay. No‐template controls were run to determine any contamination. Amplification for TaqMan probes reactions was performed in a 20μl reaction volume, using 10μl TaqMan Universal Master Mix 2X (Applied Biosystems), 2μl cDNA, and 1μl TaqMan Probe 20x. The comparative delta Ct method (also known as the 2‐ΔΔCt method) was used to calculate the relative fold change in gene expression. Data were normalized to housekeeping genes (*GAPDH* and *B2M*) and gene expression in the ventral tier of SNpc (used as control = 1).

**Table 1:** Validation of Gene Expression Using TaqMan Real‐Time Polymerase Chain Reaction

Gene	Protein	TaqMan Assay ID
*GAPDH*	Glyceraldehyde‐3‐phosphate dehydrogenase	Hs99999905_m1
*B2M*	Beta‐2‐microglobulin	Hs99999907_m1
*TH*	Tyrosine hydroxylase	Hs00165941_m1
*DAT*	Dopamine transporter	Hs00997374_m1
*VMAT2*	Vesicular monoamine transporter 2	Hs00996834_g1
*GFAP*	Glial fibrillary acidic protein	Hs00909233_m1
*PMP22*	Peripheral myelin protein 22	Hs00165556_m1
*GAD1*	Glutamate decarboxylase 1	Hs01065892_m1
*PCP4*	Purkinje cell protein 4	Hs01113637_m1
*RAB3B*	Ras‐related protein Rab‐3B	Hs01001137_m1
*HCN1*	Potassium/sodium hyperpolarization‐activated cyclic nucleotide‐gated channel 1	Hs01570432_m1
*SPARC*	Secreted protein acidic and rich in cysteine	Hs00234160_m1
*SNX8*	Sorting nexin 8	Hs01030705_m1
*MT1G*	Metallothionein‐1G	Hs01584215_g1
*ANXA1*	Annexin A1	Hs00167549_m1
*ATP13A4*	Cation‐transporting ATPase 13A4	Hs01115518_m1
*LYPD1*	Ly6/PLAUR domain‐containing protein 1	Hs00375992_m1

### Immunohistochemistry and Immunoblotting

Six‐micrometer‐thick sections of the midbrain depicting SNpc on the level of the 3rd nerve were cut from the age‐matched healthy controls (n = 5) and processed for immunohistochemistry. Following antigen retrieval with autoclave at 120°C for 10 minutes in citric buffer pH 6.0, endogenous peroxidase activity was eliminated with treatment with 3% H_2_O_2_ (in phosphate‐buffered saline) and PCP4, BAB3B, and HCN1 antibodies (dilution 1:500, see above for details) incubated overnight at 4°C. For detection, the REAL EnVision Detection System (Dako, Carpinteria, CA) was used, with diaminobenzidine as chromogen.

Two 2mm‐diameter punctures were taken from the frozen SN, 1 from the ventral and 1 from the dorsal tier, from the same healthy controls used for the RNA‐seq analysis. In addition, we dissected 2mm‐diameter punctures from the dorsal SNpc from 3 cases with incidental Lewy body disease (with Braak PD stages I–III) and 3 PD patients (with Braak PD stages V–VI).^20^ Tissue was homogenized in lysis buffer (5mM hydroxyethylpiperazine ethane sulfonic acid, pH 7.4; 320mM sucrose; 1mM ethylenediaminetetraacetic acid; 0.1% sodium dodecyl sulfate; and protease inhibitors and phosphatase inhibitors [Roche, Basel, Switzerland]) to 6% wt/vol. Following incubation on ice for 30 minutes, the homogenates were spun at 5,000rpm for 10 minutes at 4°C. The total protein amount was determined using the BCA Protein Quantification Kit (Abcam, Cambridge, UK) and adjusted to 2mg/ml. Brain samples were diluted with 0.1 × sample buffer followed by 4 parts of diluted sample combined with 1 part of Fluorescent Master Mix and heated at 95°C for 5 minutes. The denatured samples, blocking reagent, primary antibodies (PCP4, 14705‐1‐AP, Proteintech Group, Rosemont, IL; RAB3B, 15774‐1‐AP, Proteintech Group; HCN1, GTX131334, GeneTex, Irvine, CA, all with 1:50 dilution; and β‐actin ab8224, Abcam, 1:100 dilution), mouse and rabbit horseradish peroxidase–conjugated secondary antibodies, and chemiluminescent substrate were dispensed into a 384‐well plate. A biotinylated ladder provided molecular weight standards for each assay. After plate loading, the separation electrophoresis and immunodetection steps took place in the fully automated Peggy Sue (Protein Simple, San Jose, CA) capillary system.

### Functional Enrichment Analysis

Gene Ontology (GO) annotations were obtained through *biomaRt* in R from Ensembl release 85, whereas Mammalian Phenotype (MP) annotations were retrieved from the MGI Batch Query tool (accessed on December 16, 2016) using all available Ensembl gene IDs from *Mus musculus* with 1‐to‐1 ortholog correspondence to *H. sapiens*. GO enrichment analysis included terms with at least 50 genes in our background population. MP enrichment analysis included all 28 major phenotypes first, and if a major phenotypic term was significantly enriched, we performed a second enrichment analysis among more specific phenotypic terms (with a minimum of 20 genes in our background population). For the actual test, we compared the number of genes assigned to each term to an estimated expected number derived from 1,000 equally sized random samples of genes. A *p* value has been estimated based on a *z* score derived from the randomizations and further adjusted for multiple testing using the Benjamini–Hochberg method. GO terms with an FDR < 0.05 and containing at least 2 genes were deemed significantly overrepresented.

### Coexpression

Based on the expression profiles across all samples available from multiple brain structures across development in BrainSpan,^21^ we calculated the coexpression between each gene pair as the Pearson correlation between their expression levels. The average coexpression between all gene pairs in each subset of differentially expressed genes (DEGs) was measured and compared to 10,000 equally sized random samples of genes from the background population. An estimated *p* value was drawn from the randomizations by counting the number of cases in which the average coexpression was equal to or higher than that observed for each set of DEGs.

### Protein–Protein Interactions

A combined protein–protein interaction network was created based on diverse resources: BioGRID 3.4 (accessed on October 13, 2016),^22^ HitPredict (accessed on October 20, 2016), IntAct (accessed on October 11, 2016), STRING (accessed on November 29, 2016, restricted to *H. sapiens* and experimental scores >0), CORUM (accessed on October 11, 2016), and Reactome (accessed on October 11, 2016). The combined network reflected the presence of an interaction in any of the aforementioned resources, representing a total of 20,591 genes and 1,973,967 interactions, where self‐interactions were not considered. We have measured both the number of interactions and the clustering in the combined protein–protein interaction network among DEGs between ventral and dorsal neurons. To compare the observed parameters to those expected by chance, we have created 1,000 equally sized randomizations with a similar degree distribution to set of DEGs. To accomplish this, we have log2‐transformed the degree distribution of all genes in our background population, divided all genes into 20 bins, and counted the number of DEGs falling in each bin, so for each randomization an equal number of genes from each bin would be drawn. Finally, a *p* value would be estimated based on the number of cases in which the number of interactions of the clustering was either equal to or higher than that observed for each set of DEGs.

### Phenotypic‐Linkage Network

A phenotypic‐linkage network (PLN) has been used to integrate diverse functional sources of information into a single measure. From coexpression based on the BrainSpan dataset, only the top 5% of the strongest correlations were taken into account for the PLN. The combined protein–protein interaction network has also been included, as well as similarity between shared GO annotations, measured separately for biological processes, molecular function, and cellular component.^23^

## Results

Our study sought to reveal the intrinsic differences in gene expression patterns in the human healthy control individuals between the 2 neuronal populations, ventral and dorsal SNpc, known to show selective vulnerability in PD. For this, we compared the transcriptional profiles of ventral and dorsal SNpc neurons obtained from the midbrain at the level of the 3rd nerve from 7 healthy brain donors. Approximately ~100 cells from each tier were LCM, followed by RNA extraction and sequencing of each sample (see Fig 1). Samples from 3 of 7 donors were removed from further analysis, given their particularly low mapping rates and reduced library complexity. Principal component analysis based across all sequenced samples segregated along the first component samples identified and removed by their low quality.

### Transcriptional Signature of DA Neurons

**Figure 2:**
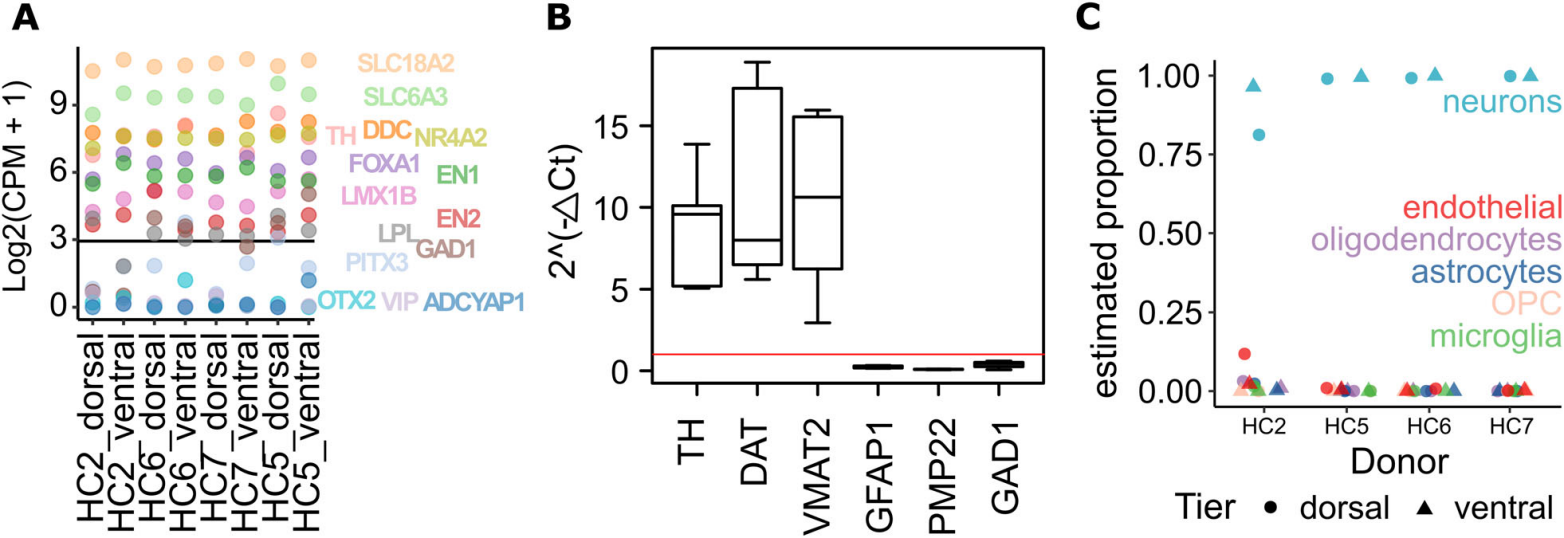
Characterization of laser capture microdissected (LCM) dopaminergic neurons. (A) Samples showed high gene expression levels (log2[CPM + 1] (CPM means counts per million)) of dopaminergic markers including dopamine transporters, enzymes involved in dopamine synthesis, and transcription factors necessary for dopaminergic specification, whereas ventral tegmental area–specific markers (*ADCYAP1*, *LPL*, *OTX2*, and *VIP*) were virtually absent. The lines denote the mean and median expression of the whole background gene population. (B) Assessment of dopamine neuron sample enrichment and purity by reverse transcribed quantitative polymerase chain reaction in LCM substantia nigra pars compacta (SNpc) neurons in relation to whole midbrain sections that have not been altered in terms of cellular content and therefore contain dopamine neurons and many other cell types. The 2‐ΔΔCt method shows increased expression of the dopaminergic genes *DAT*, *TH*, and *VMAT2* in the LCM SNpc neurons normalized to sections (value 1, *red line*), indicating dopamine neuron enrichment. A significant reduction in expression of genes for the glial fibrillary acidic protein 1 (GFAP), peripheral myelin protein 22 (PMP22) and glutamate decarboxylase 1 (GAD1) suggests isolation of a relatively pure population of dopamine neurons. (C) Proportion of neurons, astrocytes, microglia, oligodendrocytes, oligodendrocyte precursor cells (OPC), and endothelial cells estimated by MuSiC, an RNA‐sequencing deconvolution method.

We confirmed the DA identity of our samples (Fig 2A) by looking at the transcript levels of dopamine transporters (*VMAT* and *DAT*), enzymes involved in the synthesis of dopamine (*TH* and *DDC*), and transcription factors involved in dopamine specification and maintenance (*NR4A2*, *FOXA2*, *EN1*). All of them were highly expressed when compared to the average expression of all protein‐coding genes. Additionally, *LMX1B* and *EN2* were moderately expressed, whereas *PITX3* was detected just below the median of the whole gene population. Given the presence of DA neurons in the neighboring VTA, we examined the expression levels of genes reported to be specific to the VTA (*OTX2*, *ADCYAP1*, and *VIP*) and found them to be virtually undetectable.

By real‐time quantitative polymerase chain reaction (reverse transcribed‐qPCR), the relative expression levels of all dopamine neuron‐related transcripts (*TH*, *DAT*, *VMAT2*) were significantly increased in the microdissected nigral neurons compared to whole midbrain sections (see Fig 2B). This confirmed the efficiency of the LCM process in terms of its ability to quantitatively capture the targeted DA cells. Correspondingly, the expression of astroglial (*GFAP*), oligodendroglia (*PMP22*), and γ‐aminobutyric acidergic (GABAergic; *GAD1*) genes were very low, suggesting isolation of a relatively pure population of dopamine neurons. Finally, the proportion of brain cell types within our samples was estimated with MuSiC,^24^ a recent deconvolution method for bulk RNA‐seq that we applied along with single cell gene expression data from neurons, oligodendrocytes, astrocytes, microglia, and endothelial cells.^25^ This estimate supported a very high percentage of neurons within our samples (mean = 96.87%; see Fig 2C). No significant difference was found between dorsal and ventral samples (*p* > 0.05).

### DEGs between Human Ventral and Dorsal SNpc Neurons

**Figure 3:**
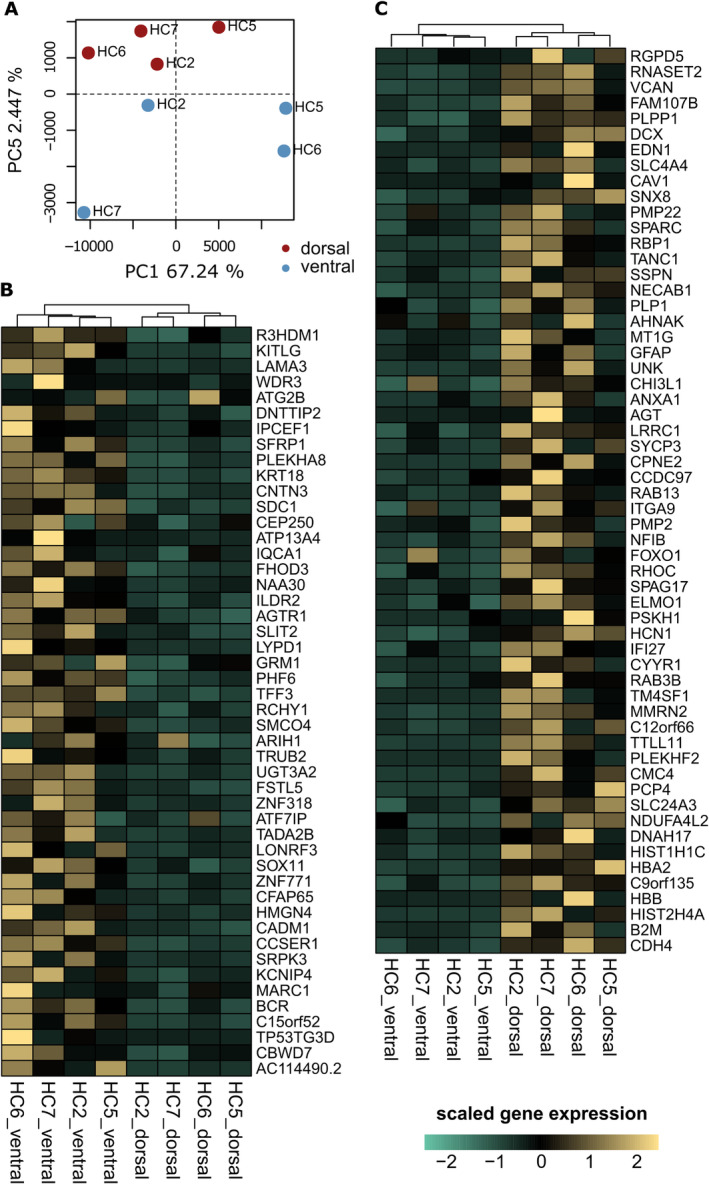
Gene expression differences between human vulnerable and resistant substantia nigra pars compacta (SNpc) neurons. (A) Principal component (PC) 5 after removal of unwanted variance factor 1 separates ventral and dorsal SNpc neurons and explains ~2.44% of the variance. HC = healthy control. (B, C) Expression patterns for genes detected differentially expressed between tiers (false discovery rate < 0.05) shown for (B) genes with significantly higher expression in ventral/vulnerable SNpc and (C) genes with significantly higher expression in dorsal/resistant SNpc. Gene expression is given in counts per million (CPM) and scaled by gene.

To obtain an unbiased set of genes with distinct patterns of expression between human ventral and dorsal SNpc neurons, we considered 14,033 protein‐coding genes remaining following the removal of outliers and genes with low expression (see Subjects and Methods). We first applied RUV analysis^18^ to remove the first factor of variation, which was associated with RIN score (Pearson correlation *r* = 0.956). Despite application of RUV, the principal drivers of variation in the data continued to be associated with technical and sample source (genotype) variation rather than anatomical localization. Nonetheless, ventral and dorsal samples segregated along the 5th component, which accounts for 2.44% of the gene expression variance (Fig 3A). We then applied DESeq2 accounting for the origin (donor) of each pair of ventral/dorsal samples. In total, 106 genes were detected as differentially expressed (FDR < 0.05), 58 of which were higher in the dorsal/resistant neurons, whereas the remaining 48 were higher in the ventral/vulnerable neurons (see Fig 3B, C). We tested whether the number of DEGs was higher than expected by chance by permuting label groups (dorsal/ventral) of samples followed by differential gene expression analysis. Among these permutations, the number of genes detected as differentially expressed was always smaller than the number obtained when the correct labels were assigned (observed = 106, permutations = 41, 67, and 25), confirming that the number of DEGs identified between tiers was higher than expected by chance.

### Validation with RT‐qPCR and Further Protein Analyses of the Top DEGs

**Figure 4:**
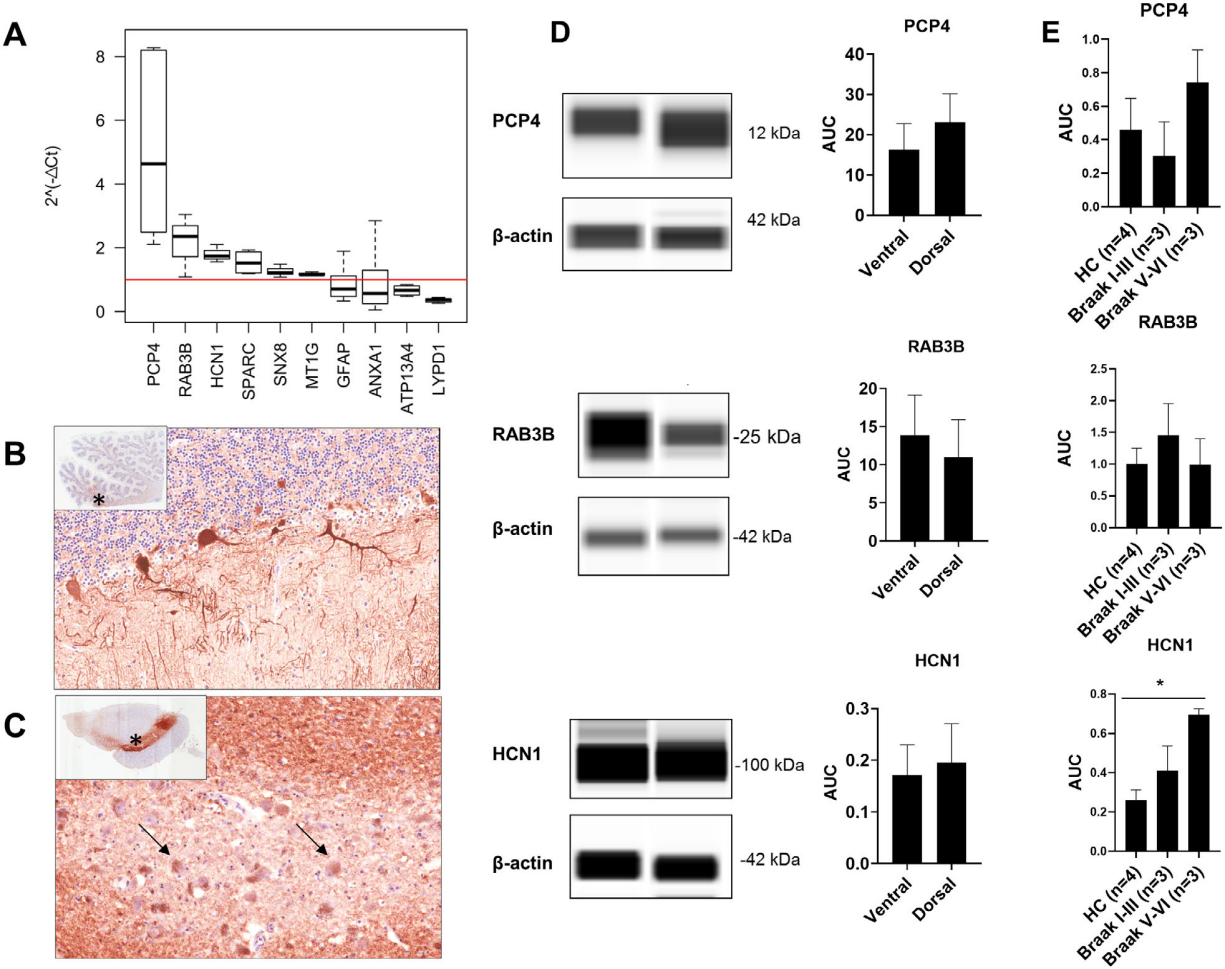
Reverse transcribed quantitative polymerase chain reaction validation and protein analysis of top differentially expressed genes. (A) Using the 2‐ΔΔCt method, increased gene expression of *PCP4*, *RAB3B*, *HCN1*, *SPARC*, *SNX8*, and *MT1G* was found in the dorsal substantia nigra pars compacta (SNpc) tier in relation to the ventral SNpc tier (marked as value 1, *red line*). *ATP13A4* and *LYPD1* were downregulated in dorsal SNpc, whereas *GFAP* and *ANXA1* showed highly variable expression between the SNpc tiers. (B, C) PCP4 immunohistochemistry strongly labeled the cell bodies and processes of the cerebellar Purkinje cells (B) and the processes around SNpc and SN pars reticulata (C), leaving devoid of the soma of the pigmented dopaminergic neurons of the SNpc *(arrows)*. Asterisks in inserts show the position of the figures at ×200 magnification. (D, E) Immunoblots and histograms (mean ± standard error of the mean) showing relative protein expression of PCP4, RAB3B, and HCN1 in the ventral versus dorsal tiers of SNpc (D) and in the dorsal SNpc between different Braak stages (E). The results are expressed as the ratio of target protein/β‐actin (internal control) in each group. AUC = area under the curve; HC = healthy controls.

Using the 2‐ΔΔCt method to determine the fold differences of relative gene expression between dorsal and ventral SNpc tiers in our top 10 DEGs largely confirmed the results from the RNA‐seq analysis (Fig 4). As predicted, RT‐qPCR analysis found *PCP4* highly increased in dorsal versus ventral SNpc neurons, followed by *RAB3B*, *HCN1*, *SPARC*, *SNX8*, and *MT1G*, whereas the expression of *ATP13A4* and *LYPD1* were decreased. However, we could not confirm by RT‐qPCR the dorsal upregulation of *GFAP* and *ANXA1* found by RNA‐seq. The PCP4 (Purkinje cell protein) immunohistochemistry showed extensive labeling of the processes in the SNpc and pars reticulata. However, we could not detect any cytoplasmic staining of the pigmented DA SNpc neurons. The RAB3B expression was also rather diffuse throughout the midbrain, whereas after trialing several HCN1 antibodies we could not detect any reliable staining in our postmortem brain tissue. Using automated quantitative Western blot, we further examined the protein levels of our top 3 DEGs in dorsal versus ventral SNpc tier (in 2mm‐diameter punctures). We found that PCP4 and HCN1 appeared to have slightly higher protein levels in the dorsal SNpc tier, similarly to their gene expression, whereas RAB3B showed higher protein levels in the ventral SNpc, in contrast to the mRNA level findings. However, the variability between punctured samples was high, and none of the differences reached statistical significance. Furthermore, we examined protein expression levels of the same 3 proteins in dorsal SNpc during the pathological progression of PD and showed that HCN1 protein expression significantly increased with Braak PD stages (analysis of variance, *p* = 0.03). The PCP4 levels appeared to increase at later Braak stages (V–VI), whereas RAB3B levels were more stable throughout the disease progression.

### Insights from Functional Analysis

As a general approach, we looked for evidence of functional associations among each group of upregulated genes (ventral/vulnerable and dorsal/resistant neurons), while controlling for the total set of genes expressed across the sampled cells. Gene pairs with coordinated expression patterns are more likely to be involved in a similar function,^26^, ^27^ and so we determined the coordinated expression pattern for each pair of DEGs with the brain across different structures and development using the BrainSpan Atlas of the Developing Human Brain.^21^ We found that the average coexpression between increased genes in the dorsal/resistant neurons is higher than we would expect from equally sized random samples of genes (empirical *p* = 0.0005), supporting convergent functionality. However, genes increased in the ventral/vulnerable neurons are not significantly coexpressed across brain tissues (empirical *p* = 0.2762).

**Figure 5:**
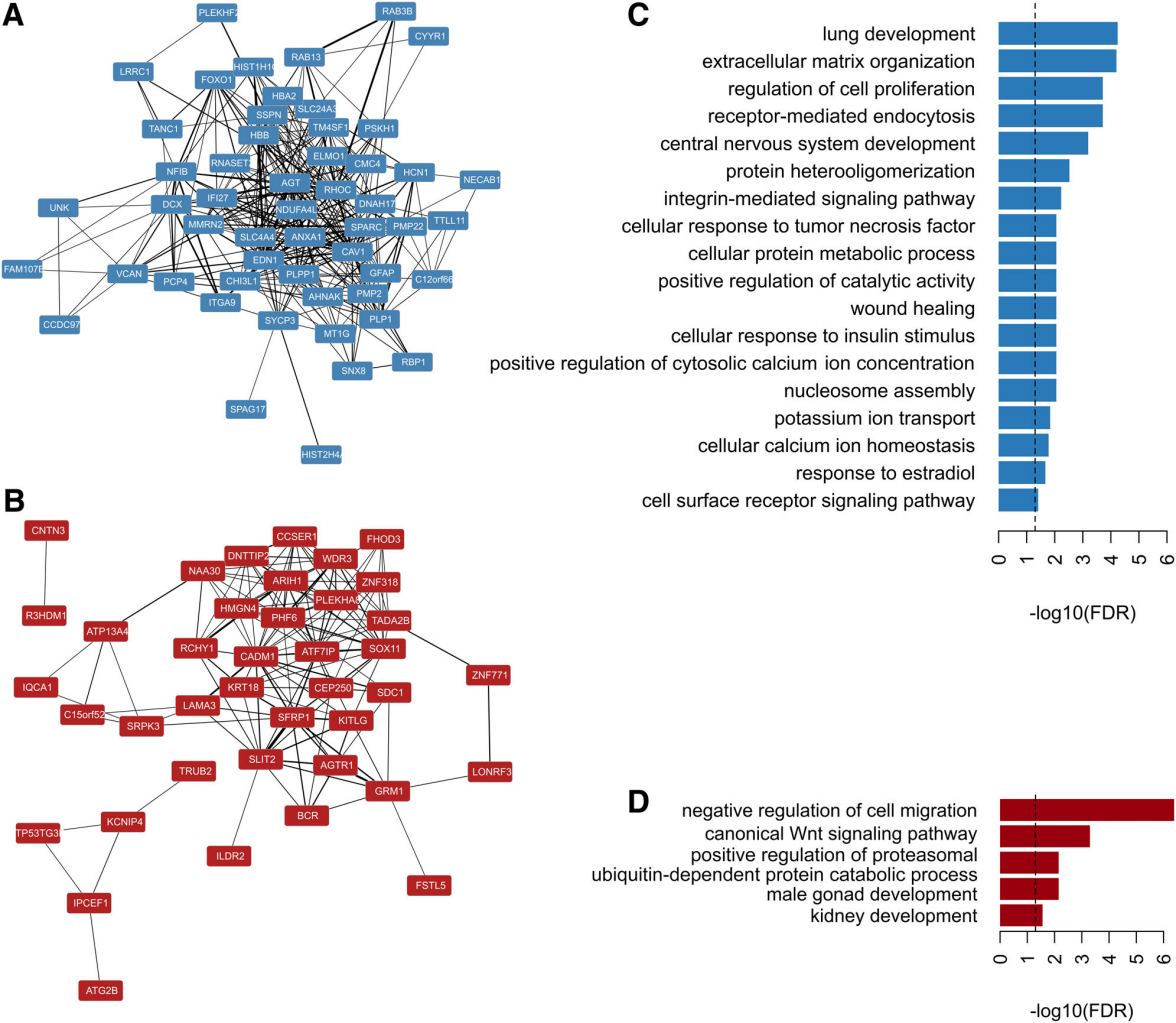
Functional convergence among increased genes in neuronal populations of each substantia nigra pars compacta (SNpc) tier. The phenotypic linkage network integrates diverse sources of functional association (gene ontology, coexpression, and protein–protein interactions) into a single measure of functional similarity between genes, where nodes represent genes and edges denote the weighted functional association between them. (A, B) Phenotypic linkage subnetwork for all increased genes (A) in the dorsal/resistant neurons and (B) in the ventral/vulnerable neurons. (C, D) Overrepresented gene ontology annotations (false discovery rate [FDR] < 0.05) for (C) genes with higher expression in dorsal/resistant SNpc and (D) for genes with higher expression in ventral/resistant SNpc. Dashed lines indicate an FDR threshold of 0.05.

Using a comprehensive integrated functional genomics approach, termed a PLN, we looked for evidence of functional convergence among DEGs. A PLN combines multiple sources of functional information (GO annotations, coexpression, and protein–protein interactions) into a single weighted measure of gene functional relatedness between all pairs of genes.^23^ To measure functional convergence, we compared the sum of the weighted edges between gene pairs in the PLN. We observed higher than expected functional convergence between genes increased in the dorsal/resistant neurons (*p* < 0.001; Fig 5A), whereas genes increased in the ventral/vulnerable neurons did not functionally converge more than expected by chance (*p* = 0.594; see Fig 5B). When considering the structure of links among DEGs within the PLN by calculating the global clustering and in comparison to random samples of genes, we noticed a higher than expected clustering for both increased genes in the dorsal/resistant (*p* = 0.033) and in the ventral/vulnerable neurons (*p* = 0.004). Overall, genes increased in the dorsal/resistant neurons have stronger links and tend to share links with neighboring genes within the PLN, whereas genes increased in the ventral/vulnerable neurons have weaker links but still share common neighbors within the PLN. Taken together, these network results provide evidence of functional clustering and thus shared functionality.

Among genes higher in the dorsal/resistant neurons, we found significantly overrepresented GO Biological Process terms *receptor mediated endocytosis* (fold overrepresentation [FOR] = 8.62), *calcium ion homeostasis* (FOR = 7.49), *potassium ion transport* (FOR = 7.43) *regulation of cell proliferation* (FOR = 7.56), *cell surface receptor signalling* (FOR = 4.53), *central nervous system development* (FOR = 7.69), *cellular response to TNF* (FOR = 8.62), and *insulin stimulus* (FOR = 7.66; see Fig 5C). By contrast, genes higher in ventral/vulnerable neurons were overrepresented in processes such as the *negative regulation of cell migration* (FOR = 14.15), *canonical Wnt signalling* (FOR = 11.49), and *positive regulation of proteasomal ubiquitin dependent protein catabolic process* (FOR = 9.47; see Fig 5D). The complete GO enrichment analysis results are given in the Supplementary Table. Overrepresentation of a unique set of specific processes was found among genes upregulated in neurons from each tier.

**Figure 6:**
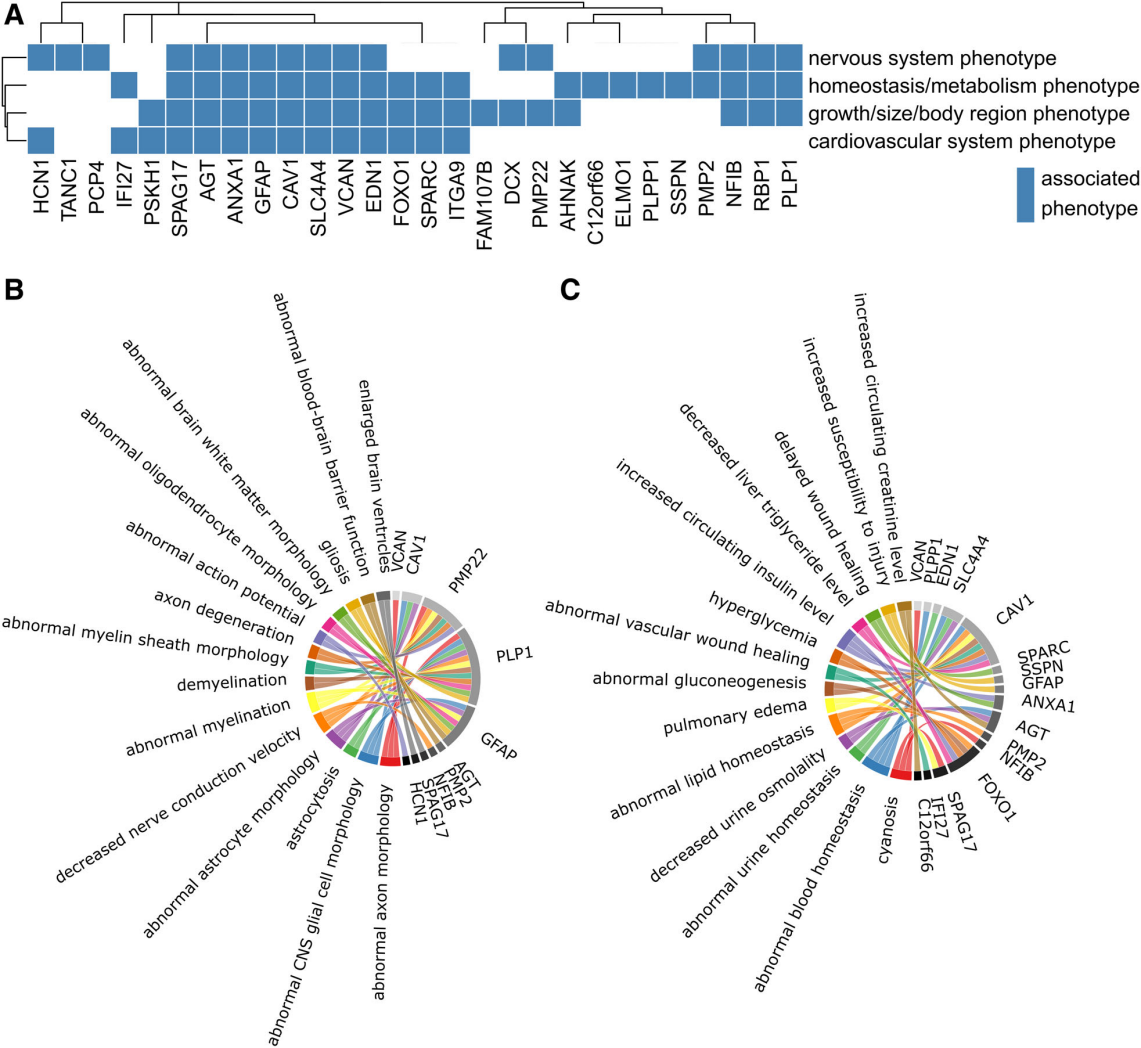
Mammalian Phenotype annotation and overrepresentation analysis for increased genes in the dorsal/resistant substantia nigra pars compacta (SNpc) neurons. (A) Genes with higher expression in dorsal/resistant neurons annotated to at least 1 overrepresented Mammalian Phenotype class (false discovery rate < 0.05). (B) Specific nervous system phenotypes overrepresented in genes with higher expression in dorsal/resistant SNpc neurons. (C) Specific homeostasis‐ or metabolism‐associated phenotypes overrepresented in genes with higher expression in dorsal/resistant SNpc neurons. CNS = central nervous system.

Additionally, we looked for any unusually overrepresented phenotypes among the unique (1:1) orthologues of these genes that had been experimentally disrupted in the mouse. Among genes increased in the dorsal/resistant neurons, we found 4 phenotypic classes overrepresented (Fig 6A), including *Nervous System* (FOR = 1.84) and *Homeostasis/Metabolism* (FOR = 1.79). Examining more specific terms within the *Nervous System* category (see Fig 6B) revealed an overrepresentation of genes whose unique mouse orthologue's disruption yields a decreased nerve conduction velocity (FOR = 22.38), abnormal myelination (FOR = 9.64), and axon degeneration (FOR = 7.75). No mouse orthologue phenotypic associations were found among genes increased in the ventral/vulnerable neurons.

### Links to Known PD Genes

The differential susceptibility to neurodegeneration between neurons from the ventral and dorsal tiers of SNpc led us to search for genes (and interactions) linked to the disease in the DEG sets. We examined the identified DEGs between the 2 neuronal populations for genes associated with familial PD, or those genes located within regions of high linkage disequilibrium (LD) to single nucleotide polymorphisms (SNPs) associated with the disease by recent PD meta‐analyses.^28^ None of the DEGs was among the familial PD genes or in high LD to associated genome‐wide association study (GWAS) SNPs. Although we did identify protein–protein interactions between DEGs and PD‐associated genes, the number of interactions was no more than we would expect by chance when compared to random genes expressed in these neuron types.

### Human to Mouse Gene Expression Comparison

**Figure 7:**
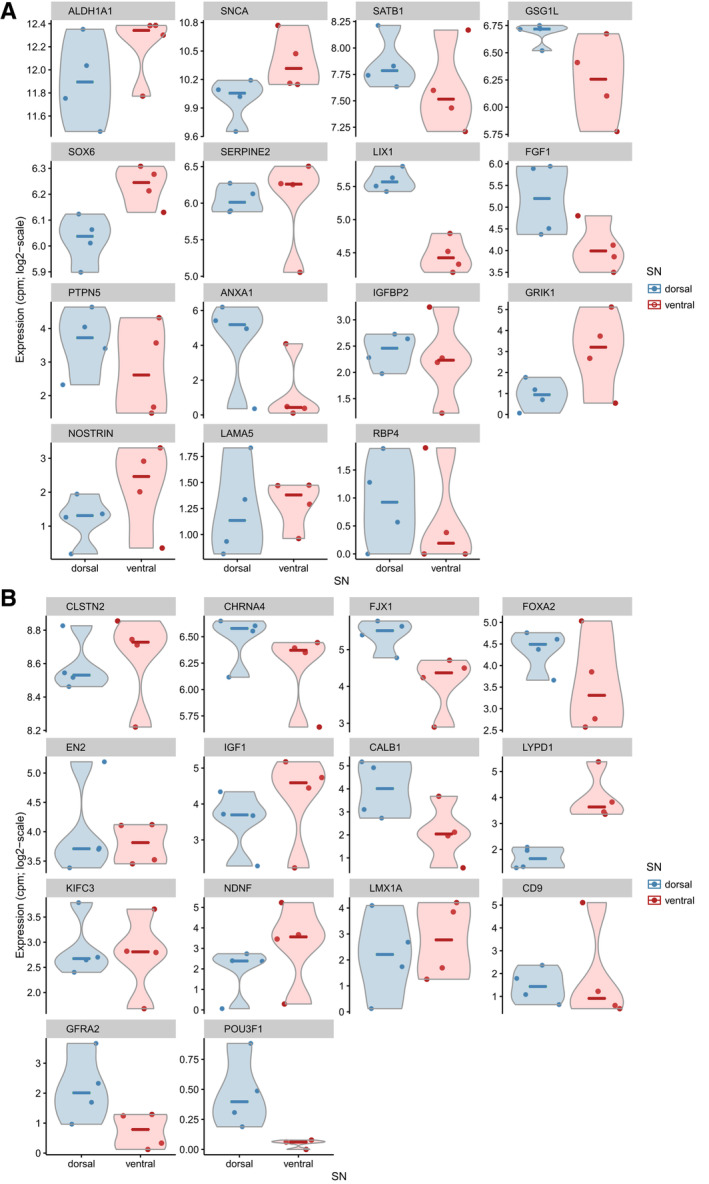
Human gene expression patterns in ventral and dorsal substantia nigra pars compacta (SNpc) neurons. (A) Genes that were expected to have higher expression in the ventral tier of SNpc according to previous mouse data also included genes (*SATB1*, *ANXA1*, *FGF1*, and *PTPN5*) that showed higher expression in the dorsal tier of SNpc in humans. (B) Likewise, genes that were expected to have higher expression in the dorsal tier of SNpc also included *LYDP1*, with higher expression in the ventral tier of SNpc in humans. CPM means Counts per million.

Mouse FACS‐sorted midbrain DA neurons have been previously distinguished based on the transcriptional profiling with an array including genes reported to be differentially expressed between SN and VTA, validated DA markers, and housekeeping genes.^14^ Two DA neuron clusters identified by Poulin et al^14^ had similar expression profiles to SNpc (DA1A‐1B) and VTA (DA2A‐2D), and further subdivision into DA1A and DA1B subtypes corresponded to the ventral and dorsal tiers of human SNpc, respectively. Furthermore, recently mouse DA neurons were classified into 5 distinct subpopulations DA‐SNC and DA‐VTA1–4 using single‐cell RNA‐seq.^15^ As the DA‐VTA1 subgroup in La Manno et al^15^ was positioned more toward the dorsal SNpc per Allen Mouse Brain Atlas, we considered this comparable to our human dorsal SNpc and the mouse DA‐SNC subgroup to be comparable to our human ventral SNpc. Thus, for gene markers reported to be different between DA1A/DA‐SNC and DA1B/DA‐VTA1 in mice, we looked at the relative expression in our human data. Some genes reported to have higher expression levels in analogous ventral SNpc mouse subpopulations (*ALDH1A1*, *SNCA*, *SOX6, GRIK1*, and *NOSTRIN)* showed similar tier‐specific directionality in humans (Fig 7A), although none of the human genes reached statistical significance. Similarly, *CHRNA4*, *FJX1*, *FOXA2, CALB1*, *GFRA2*, and *POU3F1*, with higher reported expression in analogous dorsal SNpc mouse subpopulations, also tended to have higher levels in our human dorsal SNpc neurons (see Fig 7B). However, we also identified genes with opposite trends between the species. For example, *SATB1*, *FGF1, PTPN5*, and *ANXA1* were unexpectedly more expressed in the human dorsal SNpc neurons and *LYPD1* in the ventral SNpc neurons, despite the opposite direction having been reported in mice.^14^, ^15^ Nevertheless, from all the genes compared between humans and mice, only 2 (*ANXA1* and *LYPD1*) were deemed differentially expressed in our human analysis (FDR < 0.05).

## Discussion

Our study focused on comparing the RNA‐seq transcriptomic profiles of the ventral and dorsal tiers of SNpc neurons in healthy individuals as we sought to identify intrinsic differences between the 2 neuronal populations that could inform on their differential vulnerability in the context of PD. Performing the comparison among healthy individuals aids in capturing similar numbers of neurons from both tiers; furthermore, the selection of cells is not biased toward only surviving neurons, as might occur if the same neuronal populations were compared between PD patients and healthy controls. LCM neurons were selected on the basis of their pigmentation and anatomic localization and expressed very high levels of *VMAT*, *DAT*, *TH*, and *DDC* genes, reflecting a DA profile. No significant differences in these DA marker gene levels were found between neurons from the ventral and dorsal tiers of the SNpc, supporting our aim to capture and compare similar numbers of DA neurons. It is also important that the expression levels of genes reported to be specific to the VTA (*OTX2*, *ADCYAP1*, and *VIP*) were virtually undetectable, reflecting the absence of VTA DA neurons in our samples. In addition to RNA‐seq, we validated the level of enrichment and purity of dopamine neurons by RT‐qPCR, assessing the dopamine neuron and glial/GABAergic‐specific gene expression in the LCM cells versus whole midbrain sections. However, despite the careful execution of LCM, "contamination" with non‐neuronal transcription signals (eg, *GFAP*, *PMP22*) can occur, but this is at much lower levels than what is found in the bulk tissue. However, we also confirmed a very high neuronal proportion of our sample by MuSiC,^24^, ^25^ a recent RNA‐seq deconvolution method for bulk tissue.

We found 106 DEGs between neuronal populations from the ventral and dorsal tier (FDR < 0.05), corresponding to 0.75% of our background gene population and 0.5% of all protein‐coding genes. We consider that some genes increased in the dorsal neurons (n = 58) could provide a protective effect in PD, and/or conversely, genes increased in the ventral neurons (n = 48) might confer higher vulnerability. Of particular interest in the context of neurodegeneration and PD are those genes that show abnormal phenotypes of the nervous system when the corresponding mice ortholog is disrupted, but also those directly involved in metabolic processes, given the high energy demand of DA neurons. Our study revealed a coherent group of genes increased among the dorsal/resistant neurons with functional convergence in phenotypes associated with neural function and homeostasis, many of which may be relevant to the possible protective effect of these neuronal populations in PD (see below). These genes demonstrated more coordinated temporal and spatial expression patterns across the human brain, their protein products were more likely to interact, and they presented stronger functional associations with each other than expected by chance in an integrative functional gene network. In contrast, genes increased in the more vulnerable ventral SN neurons did not exhibit strong evidence for shared functionality.

### Vesicular Trafficking

Genetic discoveries have brought defects in vesicle trafficking to the forefront of potential pathogenic players in PD.^29^ The Rab protein family enriched in neuronal synaptic vesicles plays a key regulatory role in vesicle trafficking. Rabs have been shown to be closely associated with α‐synuclein (aSyn)‐mediated pathological processes but also to interact with many PD‐related genes, and thus they could plausibly be regarded as novel biomarkers or therapeutic targets.^28^ We found 2 members of this family increased among the dorsal/resistant SNpc neurons: *RAB3B* and *RAB13*. The subfamily of RAB3 proteins in particular has been reported to have stronger gene expression in the relatively spared VTA than in the SN neurons in a human brain and elevated protein expression in the projections of the VTA in the ventromedial striatum of the rat.^11^, ^30^ When overexpressed in rat SN DA neurons, it increases the number and size of synaptic vesicles in the DA presynaptic terminals and dopamine content in the striatum.^30^ When *RAB3B* is overexpressed, it also shows a protective effect in DA neurons treated with oxidative stressor 6‐hydroxydopamine and the proteasome inhibitor. Conversely, when *RAB3B* is knocked down by siRNA, DA neurons show an increased vulnerability to both toxins.^29^ A protective effect of RAB13 on the other hand involves the clearance of aSyn inclusions through endocytic recycling and rescued aSyn‐induced toxicity.^31^ Furthermore, several members of the RAB family including RAB3 get phosphorylated by LRRK2,^32^ whereas RAB13 phosphorylation occurs in response to PINK1 activation.^33^

In addition to Rab genes, *SNX8* (sorting nexin 8) and *ANXA1* (annexin A1), genes involved in intracellular trafficking, were higher among dorsal/resistant SNpc neurons. *SNX8* is a key element of retromer‐mediated endosomal protein sorting, also emerging as important cellular machinery in PD.^34^ *ANXA1* is a key mediator of endoplasmic reticulum (ER) contact sites required for the ER‐derived cholesterol transport to endosomes.^35^

### Ion Transport and Homeostasis

Another shared feature of vulnerable SNpc neurons is their distinctive physiology of autonomous pacemaking activity that mainly relies on Ca^2+^ channels.^36^ However, when these channels are knocked down, a reversal to a more “juvenile” pacemaking activity dependent on Na^+^ and hyperpolarization‐activated cyclic nucleotide gated (HCN) potassium channels can protect neurons from methylphenyltetrahydropyridine (MPTP)‐induced cell death.^37^ There are 4 HCN genes in mammals (*HCN1*–*HCN4*), each of them showing different biophysical properties. Although *HCN4* has been reported to have higher transcript abundance in rodent SNpc neurons,^38^ we identified higher *HCN1* expression in dorsal/resistant SNpc neurons in humans. *HCN1* has been found to be higher with age in the MitoPark mouse, a genetic model of PD with disrupted mitochondrial function, perhaps as a compensatory effect to sustain firing rates and neural function.^39^ Our study also identified the expression of *SLC4A4* (Na^+^‐coupled acid‐base transporter), *EDN1* (endothelin‐1),^40^ and *PCP4* (Purkinje cell protein 4) as higher in the dorsal SNpc neurons, all genes that maintain Ca^2+^ homeostasis and thus may be protective. *PCP4* (also known as PEP‐19) in particular regulates Ca^2+^ binding to calmodulin, and the levels of transcript and protein of PEP‐19 are reduced in striatum of the MPTP mouse model of PD.^41^ In addition, we found higher expression of *ATP13A4*, cation‐transporting, P5‐type adenosine triphosphatase, among the ventral SNpc neurons. Mutations in this gene have been implicated mainly in the developmental disorders and have been shown to reduce calcium transport in vitro.^42^ The related family gene *ATP13A2* is actually PARK9 locus, linked to autosomal recessive familial parkinsonism,^43^ but we found no differences in its expression between the SNpc tiers.

### Oxidative Stress

Although oxidative stress processes were not directly overrepresented among the increased genes, genes related to oxidation such as *HBB* and *HBA2* (hemoglobin subunit β/α2) were higher among the dorsal/resistant SNpc neurons. Interestingly, apart from binding to O_2_ and CO_2_ in blood, hemoglobin is also present in SNpc DA neurons in mice and humans. It may act as storage for oxygen, providing homeostatic mechanisms for SNpc DA neurons that have an exceptionally high energy requirement.^44^ Furthermore, double‐labeling immunofluorescence has shown reduced levels of hemoglobin specifically in the neurons with aSyn‐immunopositive Lewy bodies.^45^ In addition, the expression of *MT1G* (metallothioneins), a free radical scavenger, was also higher among the dorsal SNpc neurons and has been shown to provide a neuroprotective effect in the rotenone mouse model of PD.^46^ The increased *SPARC* (secreted protein acidic and rich in cysteine) gene in dorsal SNpc neurons was recently linked to PD by in silico study that identified novel proteins involved in Cu and Fe metabolism.^47^

One important goal of our human‐specific transcriptomic profiling of selectively vulnerable SNpc populations was to analyze the possible links to known PD genes. Unexpectedly, we did not find any overlap between our DEG and PD familial genes or those genes located within regions of high LD to SNPs associated with the disease by recent PD meta‐analyses.^28^ However, we did find 4 increased genes that have been identified as GWAS hits of modest significance for sporadic PD by other smaller studies—3 higher among the dorsal SNpc neurons: *MMRN2* (multimerin 2),^48^ *ANXA1*,^49^ and *B2M* (beta‐2‐microglobulin)^50^; and 1 higher among the ventral SNpc neurons: *AGTR* (angiotensin II receptor type 1).^51^

Although RNA‐seq is a very powerful technique, some technical artifacts may be present in the data (eg, starting concentrations or cDNA quality), and validation with an independent technique is therefore advisable using different biological replicates from the same population. The RT‐qPCR validation of our top 10 DEGs largely confirmed the RNA‐seq results, but not always; for example, GFAP, being one of the most abundant proteins of the brain, is likely to be a result of contamination by the LCM process itself. However, 3 of our DEGs (*PCP4*, *BAB3B*, and *HCN1*) showed clearly higher gene expression in the dorsal versus ventral SNpc neurons. Thus, we wanted to further examine their protein levels, although it is important to note that these analyses are entirely observational rather than validating our RNA‐seq results as such. The relationship between mRNA and protein is complex, as the steady‐state transcript abundances only partially predict the protein levels, and processes downstream of transcription play a strong regulatory role.^52^ Although mass spectrometry–based proteomic analysis of neurons isolated from postmortem human brain by LCM is possible,^53^ this was beyond the scope of our current study. Therefore, we examined the protein levels in situ using immunohistochemistry but also immunoblotting the 2mm punctures from both ventral and dorsal SNpc tiers. Furthermore, we were interested to see whether we could observe any changes in the protein levels during the progression of PD pathology (ie, in different Braak stages).

The PCP4 immunohistochemistry showed extensive labeling of the processes throughout the SNpc devoid of any clear cytoplasmic staining of the pigmented DA SNpc neurons. In the human brain, PCP4 immunoreactivity is mainly found in the processes of basal ganglia and substantia nigra, and in the cell bodies of thalamic nuclei, dentate gyrus, and Purkinje cells.^54^ Thus, our RNA‐seq signal with *PCP4* likely originates from the processes that are inevitably included in the LCM procedure, the epitope of our antibody does not recognize PCP4 in the cell body (eg, similar to neurofilament staining), or increased mRNA does not manifest as protein change detectable with immunohistochemistry. Similarly, the RAB3B proteins^30^ are mainly enriched in the presynaptic terminals, and we detected rather diffuse reactivity throughout the midbrain. Although the variability between the 2mm puncture samples was high, PCP4 and HCN1 appeared to have slightly higher protein levels in the dorsal SNpc, similar to their gene expression. On the other hand, RAB3B showed a tendency to higher protein levels in the ventral SNpc, in contrast to its mRNA, highlighting further that mRNA and protein are not necessarily congruent. Finally, we showed that HCN1 protein expression in the dorsal SNpc increased with progression of PD pathology, suggesting the possibility of a compensatory effect similar to that observed in the MitoPark mouse,^39^ but this needs further investigation.

Our comparisons of gene expression variation in human ventral and dorsal SNpc DA neurons to analogous groups of DA1A/DA‐SNC and DA1B/DA‐VTA1 characterized in mice^14^, ^15^ identified several genes with similar orientation in the expression pattern across the tiers. However, none of these genes was found to be significantly differentially expressed in humans. In our study, only *LYPD1* and *ANXA1* showed significant differences in expression, and they were increased in the opposite direction across the tiers between the 2 species. Such differences found between humans and mice justify the importance of a human‐focused understanding of the intrinsic difference between the different populations of DA neurons.

This study underscores the value of human postmortem transcriptome studies, which to date are hampered by the scarcity of suitable tissue resources. Future studies would benefit from higher sample numbers and single‐nuclei sequencing instead of LCM to avoid cell type contamination issues. However, nuclei sequencing is limited to capturing only a fraction of the cellular mRNA population. Human‐focused omics atlases such as that described here provide important references for future research.

## Author Contributions

Study conception and design: R.W.‐M., C.W., L.P.; data acquisition and analysis: J.M.‐S., T.I., I.P., L.P.; drafting the manuscript and figures: J.M.‐S., T.I., L.P.

## Potential Conflicts of Interest

Nothing to report.

## Supporting information


**Appendix** **S1:** Supporting information
